# Plerixafor stem cell mobilization in Japanese children: A post‐marketing study

**DOI:** 10.1111/ped.15106

**Published:** 2022-04-09

**Authors:** Hiroaki Goto, Rie Kanamori, Satoshi Nishina, Takashi Seto

**Affiliations:** ^1^ Division of Hematology/Oncology Kanagawa Children’s Medical Center Yokohama Japan; ^2^ Sanofi Genzyme Medical Oncology Medical Sanofi K.K. Tokyo Japan; ^3^ Medical Affairs Post‐Authorization Regulatory Studies Sanofi K.K. Tokyo Japan

**Keywords:** autologous stem cell transplantation, children, plerixafor, post‐marketing surveillance, solid tumors

## Abstract

**Background:**

Plerixafor is approved in Japan for hematopoietic stem cell mobilization prior to autologous transplant, but limited data are available on the use in children. This study evaluates the safety and effectiveness of plerixafor in Japanese children aged <15 years.

**Methods:**

A multicenter, post‐marketing surveillance study was conducted in Japan to evaluate the safety and effectiveness of plerixafor in routine clinical practice. This subgroup analysis examined the safety and effectiveness of plerixafor administered as a once‐daily, subcutaneous injection in children aged <15 years. The primary effectiveness outcome was the proportion of patients with 2 × 10^6^ cells CD34+ cells/kg collected via apheresis within 4 days.

**Results:**

Eighteen patients with solid tumors were included in this analysis; (median age 6.0 years, range, 1–13 years). In addition to granulocyte colony‐stimulating factor, all patients had received chemotherapy immediately prior to plerixafor administration. The mean (SD) daily dose of plerixafor was 0.24 (0.01) mg/kg. Seven of the 18 patients (38.9%) developed adverse drug reactions (ADRs), all occurring in patients aged ≥6 years and weighing ≥16 kg. The most common ADRs were pyrexia (*n* = 4), vomiting (*n* = 3), nausea (*n* = 2), and abdominal pain (*n* = 2). Twelve patients (66.7%) achieved a CD34+ cell count ≥2 × 10^6^ cells/kg within 4 days after the start of plerixafor administration.

**Conclusions:**

The results provide an encouraging sign that plerixafor 0.24 mg/kg may be safe and effective in pediatric patients in routine clinical practice in Japan, but further research in larger studies is needed.

Autologous peripheral blood stem cell transplantation (A‐PBSCT), which is performed to support myeloabalative treatments for various malignancies, requires the mobilization of hematopoietic stem cells (HSCs) from the bone marrow to the peripheral circulation, where the peripheral blood stem cells are collected via apheresis.^1^, ^2^ Mobilization is accomplished through administration of hematopoietic growth factors, such as granulocyte colony‐stimulating factor (G‐CSF) alone or in combination with chemotherapy.^1^, ^2^, ^3^, ^4^, ^5^ Unfortunately, in response to standard G‐CSF‐based mobilization regimens alone, patients may fail to generate the minimum level of CD34+ stem cells needed for A‐PBSCT.^2^, ^6^, ^7^, ^8^ Poor mobilizers may require repeated mobilization attempts, which places them at risk of disease progression and mortality^9^, ^10^ as well as requiring additional healthcare and resource utilization.^4^, ^11^, ^12^ If repeated mobilization attempts fail, patients may require bone marrow harvest or an allogeneic transplant, which is a more complex procedure with higher morbidity and mortality than autologous transplant.^13^, ^14^

Plerixafor is a small‐molecule CXCR4 chemokine receptor antagonist that is used in combination with G‐CSF to augment HSC mobilization in patients undergoing A‐PBSCT. Plerixafor prevents HSCs from binding to bone marrow stromal cells by reversibly inhibiting the interaction of HSC CXCR4 receptors with stromal cell CXCL12 ligands.^15^, ^16^ As a result, HSCs no longer receive the CXCL12‐mediated retention signal, allowing them to escape the usual hematopoietic maturation pathway and enter the peripheral circulation.^15^ Plerixafor is approved in the United States and European Union (EU) for use in combination with G‐CSF for the mobilization of autologous stem cells.^16^, ^17^ Approval was based on clinical data from international phase III studies in adult patients (≥18 years) with non‐Hodgkin lymphoma (NHL) or multiple myeloma (MM). These studies showed a significant increase in the proportion of patients who achieved target CD34+ cell levels in ≤4 days of apheresis when treated with plerixafor compared with G‐CSF alone.^18^, ^19^ Plerixafor has also received regulatory approval in Japan for HSC mobilization in combination with G‐CSF on the basis of these studies^18^, ^19^ and two phase II studies conducted in adult Japanese patients with NHL or MM.^20^, ^21^ In the Japanese studies, substantially higher proportions of patients receiving plerixafor plus G‐CSF achieved CD34+ cell target levels in ≤2–4 days of apheresis compared with patients receiving G‐CSF alone.^20^, ^21^

While the efficacy and safety of plerixafor is well established in adults, relatively limited data are available on the use of plerixafor for HSC mobilization in children, particularly Asian children. Direct extrapolation of adult data is not possible because of inherent differences between adult and pediatric populations, including different underlying malignancies requiring different chemotherapy and radiotherapy regimens.^14^ The international phase I/II MOZAIC study was conducted in children (1 to <18 years) with solid tumors, including neuroblastoma, sarcoma, medulloblastoma, or lymphoma, and found that plerixafor plus standard mobilization (G‐CSF ± chemotherapy) significantly increased the proportion of patients achieving a doubling of the peripheral CD34+ cell count in the 24 h before first apheresis compared with standard mobilization alone.^22^ This study, which was conducted mainly in Europe, determined that the optimal pediatric dose of plerixafor is the same as that recommended for adults (0.24 mg/kg administered 8–12 h before apheresis),^22^ and led to approval of plerixafor for pediatric use in the EU.^14^ Plerixafor is already used in pediatric patients at 0.24 mg/kg according to the adult dose, but its safety and efficacy have not been fully tested.

In order to confirm the safety and effectiveness of plerixafor in Japanese patients, a post‐marketing surveillance (PMS) study that included children was undertaken in Japanese patients who were undergoing A‐PBSCT and received plerixafor for HSC mobilization.^23^ This report describes the safety and efficacy data for plerixafor in a subgroup of pediatric patients from a PMS study.

## Methods

### Design and patients

Details of the study design have been previously published.^23^ Briefly, this was an observational, multicenter, PMS study conducted in patients in Japan who received plerixafor for HSC mobilization prior to A‐PBSCT. The surveillance period was between February 2017 and March 2019, with patient registration undertaken between February and December 2017.

In patients receiving G‐CSF, plerixafor was administered subcutaneously at the recommended adult dose of 0.24 mg/kg (dosage calculated on actual body weight) once daily until the completion of PBSC collection with apheresis. Eligible patents were observed from the start of the plerixafor treatment until 30 days after the first dose, or the day prior to radiotherapy, or chemotherapy, whichever occurred first.

The study was conducted in accordance with Good Post‐Marketing Study Practice requirements defined by the Japanese Ministry of Health, Labor and Welfare (Ministry of Health, Labour and Welfare, Ministerial Ordinance on Good Post‐marketing Study Practice for Drugs. No. 171, December 20, 2004). Under these regulations, informed consent from individual patients was not required. The study protocol was reviewed and approved by the institutional review boards of each institution.

This subgroup analysis included patients aged <15 years old.

### Data collection and assessments

Physicians recorded patient data on case report forms or using an electronic data capture system.

Baseline data were collected included disease characteristics, performance status, complications, prior treatments, concomitant medications, renal and hepatic function, white blood cell count, creatinine clearance, and CD34+ cell count in peripheral blood samples obtained before apheresis. The average plerixafor dose, the total number of plerixafor doses, and the number of days G‐CSF was used were also recorded.

Adverse events (AEs), serious AEs, and adverse drug reactions (ADRs) were recorded and categorized by system organ class (SOC) and preferred term (PT) using MedDRA/J version 22.1 (Medical Dictionary for Regulatory Activities of Japanese Maintenance Organization [MedDRA/J], Tokyo, Japan). ADRs were AEs for which a causal relationship with plerixafor could not be ruled out. ADRs of special interest, including allergic and hypersensitivity reactions, leukocytosis, thrombocytopenia, interstitial lung disease, myocardial infarction, tumor cell mobilization, and splenomegaly/splenic rupture, were also evaluated. Vital signs and laboratory parameters were recorded.

The primary effectiveness outcome was the proportion of patients with a CD34+ cell count of 2 × 10^6^ cells/kg within 4 days of apheresis. The number of days of apheresis required to collect 2 × 10^6^ CD34+ cells/kg was also determined.

### Statistical analysis

The safety analysis group comprised all pediatric patients who received plerixafor treatment and had no registration violations. The effectiveness analysis group included all pediatric patients in the safety analysis group who received plerixafor according to the approved dosage and administration schedule and had CD34+ data available.

Baseline demographics were summarized using descriptive statistics. Continuous variables were analyzed using mean ± SD, and median (range) and categorical variables were summarized using the number and proportion. The frequency of AEs was summarized descriptively overall and for each individual event (by SOC and PT).

All statistical analyses were conducted using SAS software, version 9.4 (SAS Institute Inc., Cary, NC, USA).

## Results

### Patient disposition

**Fig. 1:**
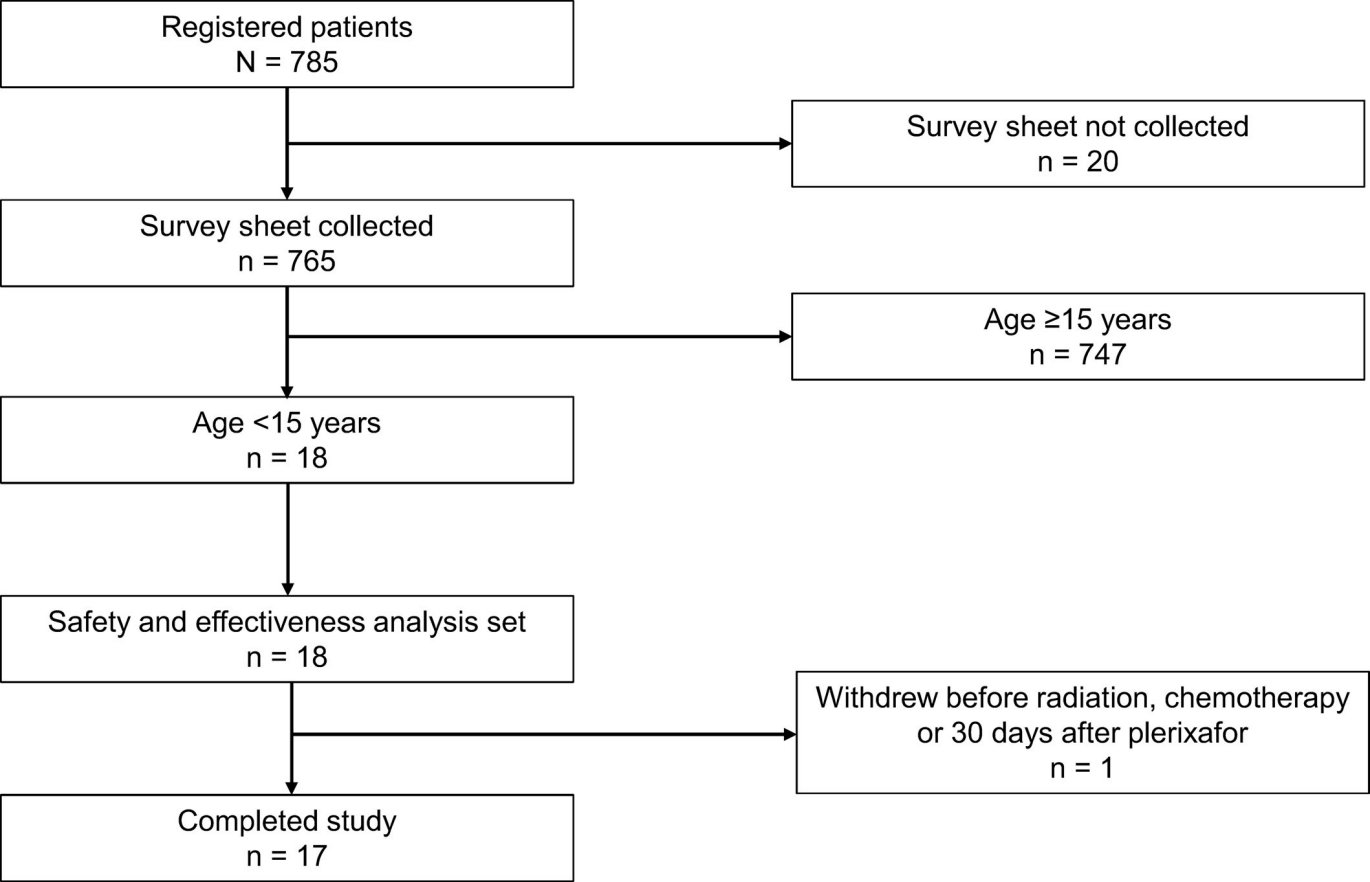
Flow chart of patient disposition.

Eighteen patients at 10 centers (6 boys and 12 girls) aged <15 years received plerixafor and all 18 patients were included in the safety and effectiveness analysis groups (Fig. 1). Seventeen patients were observed until the initiation of chemotherapy (*n* = 16; 88.9%) or 30 days after plerixafor administration (*n* = 1; 5.6%). One patient (5.6%) discontinued the study before the protocol‐defined endpoint because they required closure of a cerebrospinal fluid leak caused by their primary disease.

Patients were aged between 1 and 13 years (median 6 years), including seven patients aged <6 years (median 3 years) and 11 patients aged 6 to <15 years (median 8 years; Table 1). The most common diagnosis was neuroblastoma (*n* = 8; 44.4%), followed by medulloblastoma (*n* = 5; 27.8%), Ewing sarcoma (*n* = 2; 11.1%), and germ cell tumor (*n* = 2; 11.1%). One patient (5.6%) had rhabdomyosarcoma. Most patients (*n* = 16; 88.9%) had an Eastern Cooperative Oncology Group (ECOG) performance status of 0 or 1, but two patients (11.1%) had an ECOG performance status of 3. None of the patients had hepatic or renal dysfunction at baseline. White blood cell count was unknown in 17 of the 18 patients. Fifteen patients (83.3%) received a single injection of plerixafor and three patients (16.7%) received two injections. The mean ± SD plerixafor dose was 0.24 ± 0.01 mg/kg, in accordance with the prescribing information. Patients were treated with G‐CSF for ≥4 days and all patients received chemotherapy before administration of plerixafor.

**Table 1:** Patient baseline demographic and clinical characteristics

	Overall safety analysis group (*n* = 18)	Age <6 years (*n* = 7)	Age 6 to <15 years (*n* = 11)
Age, years, mean ± SD	6.5 ± 3.8	2.7 ± 1.1	8.9 ± 2.7
Sex *n* (%)			
Male	6 (33.3)	2 (28.6)	4 (36.4)
Female	12 (66.7)	5 (71.4)	7 (63.6)
Body weight (kg), mean ± SD	21.1 ± 9.2	13.4 ± 2.9	26.0 ± 8.4
Diagnosis, *n* (%)			
Neuroblastoma	8 (44.4)	6 (85.7)	2 (18.2)
Medulloblastoma	5 (27.8)	0	5 (45.5)
Ewing's sarcoma	2 (11.1)	0	2 (18.2)
Germ cell tumor	2 (11.1)	0	2 (18.2)
Rhabdomyosarcoma	1 (5.6)	1 (14.3)	0
ECOG PS at study drug initiation, *n* (%)			
0	7 (38.9)	2 (28.6)	5 (45.5)
1	9 (50.0)	5 (71.4)	4 (36.4)
2	0	0	0
3	2 (11.1)	0	2 (18.2)
4	0	0	0
Chemotherapy prior to study drug administration, *n* (%)	18 (100.0)	7 (100.0)	11 (100.0)
Previous collection of hematopoietic stem cells, *n* (%)			
No	7 (38.9)	2 (28.6)	5 (45.5)
Yes	11 (61.1)	5 (71.4)	6 (54.5)
Daily dose of plerixafor, (mg/kg), mean ± SD	0.24 ± 0.01	0.24 ± 0.01	0.24 ± 0.01
Number of administrations of plerixafor, mean ± SD	1.2 ± 0.4	1.1 ± 0.4	1.2 ± 0.4
Number of administrations of plerixafor, category, *n* (%)			
1	15 (83.3)	6 (85.7)	9 (81.8)
2	3 (16.7)	1 (14.3)	2 (18.2)
Total number of days of G‐CSF administration, category, *n* (%)			
≤3	0	0	0
4	6 (33.3)	3 (42.9)	3 (27.3)
≥5	12 (66.7)	4 (57.1)	8 (72.7)
Number of days of administration of G‐CSF prior to plerixafor administration, category, *n* (%)			
1–3	0	0	0
4	1 (5.6)	0	1 (9.1)
5	6 (33.3)	3 (42.9)	3 (27.3)
≥6	11 (61.1)	4 (57.1)	7 (63.6)
CD34+ cell count prior to apheresis, cells/μL, category, *n* (%)			
0–<5	2 (11.1)	0	2 (18.2)
5–<10	1 (5.6)	1 (14.3)	0
10–<20	0	0	0
≥20	4 (22.2)	2 (28.6)	2 (18.2)
Not implemented	11 (61.1)	4 (57.1)	7 (63.6)

ECOG PS, Eastern Cooperative Oncology Group Performance Status; G‐CSF, granulocyte stimulating factor; WBC, white blood cell.

### Safety

Seven of the 18 pediatric patients (38.9%) developed AEs, all of which were considered to be ADRs (Table 2) and all occurring in the subgroup of patients aged 6 to <15 years and weighing ≥16 kg. ADRs occurred in three patients with medulloblastoma, two patients with Ewing sarcoma, and two patients with a germ cell tumor. Two patients (11.1%) developed serious ADRs: hypoxia in one patient and nausea/vomiting in another. Both these events resolved without sequelae. The most common types of ADR in children by SOC were gastrointestinal ADRs (*n* = 4), general disorders and administration site conditions (*n* = 4). The most common ADRs by PT were pyrexia (*n* = 4; 22.2%), followed by vomiting (*n* = 3; 16.7%), nausea (*n* = 2; 11.1%), and abdominal pain (*n* = 2; 11.1%). One patient each developed diarrhea, frequent bowel motions, oral hypoesthesia, abnormal hepatic function, back pain, hypoxia, and inflammation.

**Table 2:** Summary of adverse drug reactions reported in children during the survey period

Adverse drug reactions, *n* (%)	Overall safety analysis set (*n* = 18)	Age <6 years (*n* = 7)	Age 6 to <15 years (*n* = 11)
Any ADRs	7 (38.9)	0	7 (63.6)
Any serious ADRs	2 (11.1)	0	2 (18.2)
ADRs occurring in at least one patient by PT			
System organ class			
Preferred term			
General disorders and administration site conditions	4 (22.2)	0	4 (36.4)
Pyrexia	4 (22.2)	0	4 (36.4)
Inflammation	1 (5.6)	0	1 (9.1)
Gastrointestinal disorders	4 (22.2)	0	4 (36.4)
Vomiting	3 (16.7)	0	3 (27.3)
Abdominal pain	2 (11.1)	0	2 (18.2)
Nausea	2 (11.1)	0	2 (18.2)
Diarrhea	1 (5.6)	0	1 (9.1)
Frequent bowel movements	1 (5.6)	0	1 (9.1)
Hypoesthesia oral	1 (5.6)	0	1 (9.1)
Respiratory, thoracic and mediastinal disorders	1 (5.6)	0	1 (9.1)
Hypoxia	1 (5.6)	0	1 (9.1)
Hepatobiliary disorders	1 (5.6)	0	1 (9.1)
Hepatic function abnormal	1 (5.6)	0	1 (9.1)
Musculoskeletal and connective tissue disorders	1 (5.6)	0	1 (9.1)
Back pain	1 (5.6)	0	1 (9.1)

Coded using MedDRA/J version 22.1 (Medical Dictionary for Regulatory Activities of Japanese Maintenance Organization [MedDRA/J], Tokyo, Japan).

ADRs, adverse drug reactions; PT, preferred term.

No ADRs of special interest were reported during the survey period.

### Effectiveness

Overall, 12 of the 18 patients (66.7%) achieved a CD34+ cell count of 2 × 10^6^ cells/kg within 4 days of apheresis, including five of seven patients (71.4%) aged <6 years and weighing <16 kg, and seven of 11 patients (63.6%) aged 6 to <15 years and weighing ≥16 kg. The primary effectiveness endpoint was met in five of eight patients with neuroblastoma (62.5%), four of five patients with medulloblastoma (80.0%), one of the two patients with Ewing's sarcoma (50.0%), one of the two germ cell tumor patients (50.0%), and in the one patient with rhabdomyosarcoma (100.0%).

In the 12 patients who met the primary effectiveness target, 2 × 10^6^ CD34+ cells/kg was reached after 1 day of apheresis in 10 patients and after 2 days of apheresis in two patients. The target was reached after 1 day of apheresis in all five of the responding patients aged <6 years and weighing <16 kg. In the seven responding patients aged 6 to <15 years and weighing ≥16 kg, the target was reached after 1 day of apheresis in five patients and after 2 days in two patients.

## Discussion

This subgroup analysis of a PMS study supports the safety and effectiveness of plerixafor 0.24 mg/kg for HSC mobilization in Japanese children with solid tumors who are scheduled to undergo A‐PBSCT. This is the first study of plerixafor in a Japanese pediatric population.

The overall results from the study indicated that patients aged <15 years were more likely to experience ADRs compared with those aged ≥15 years (*P* = 0.0005).^23^ The reason for this is not clear; however, the rate of ADRs in patients aged <15 years in this study was similar to the procedure‐related AE rate reported previously among children undergoing PBSC harvests using protocols that did not include plerixafor.^24^ It was also similar to the rate among Japanese adolescents and young adult patients aged 15 to 29 years (data not shown) in the wider population of the current PMS study. The chemotherapy for pediatric solid tumors prior to PBSC harvest is usually intensive, which probably contributes to the high rate of ADRs seen in this population. Our study had no control group, so it is impossible to determine which AEs resulted from plerixafor and which were caused by chemotherapy or other treatments.

In the phase I/II MOZAIC study in pediatric patients, treatment‐emergent AEs occurred in 76.7% of 30 patients in the plerixafor treatment arm and 66.7% of 15 patients in the standard therapy arm.^22^ Compared with our study, in which 38.9% of the 18 pediatric patients receiving plerixafor developed ADRs, the MOZAIC study showed a low rate of treatment‐related AEs with plerixafor (13.3%) and none with standard mobilization.^22^ This is somewhat unexpected because post‐marketing analyses often report a lower rate of AEs than clinical trials do, since post‐marketing research relies on the judgment of the attending physician, whereas clinical trials use protocol‐defined AE assessment. Patient age may have contributed to the difference in ADR incidence between the current PMS study and the MOZAIC study. We found that ADRs occurred only in patients aged ≥6 years and weighing ≥16 kg, but the MOZAIC study included a higher proportion of plerixafor‐treated patients aged <6 years (53.3%) than our PMS study, in which 38.9% of patients were aged <6 years and weighed <16 kg. All seven patients with ADRs in our study had tumor types that were not present in the subgroup of children aged <6 years (Ewing sarcoma, germ cell tumor, medulloblastoma), so the relatively high incidence of ADRs observed in older children may reflect different tumor types and treatment histories, including previous chemotherapy cycles or radiotherapy. However, another possible reason for the higher rate of ADRs in patients aged 6–15 years is that ADRs may be overlooked in infants and young children who are less able to communicate.

In a report of plerixafor use in six Korean children aged 6–15 years, Hong *et al*. described spontaneous pneumomediastinum at 11 and 56 days after plerixafor in two children (aged 10 and 11 years) with medulloblastoma who had previously received thoracic irradiation.^25^ Their condition deteriorated to respiratory failure and both patients died at 89 and 102 days, respectively, after plerixafor administration, with pathologic findings consistent with diffuse alveolar damage. In contrast to these findings, serious ADRs occurring in two patients in our population (hypoxia and nausea/vomiting) resolved without sequelae. The ADRs observed in our study were considered consistent with the known safety profile of plerixafor in adults and children, but as cautioned by Hong *et al*.,^25^ unexpected complications may occur in susceptible patients.

The low rate of serious ADRs in our study is consistent with the MOZAIC study, in which all serious AEs were attributed to the effects of mobilizing chemotherapy^22^ as well as other studies describing real‐world use of plerixafor in children.^26^, ^27^, ^28^ Case reports of plerixafor use in children have mostly reported no drug‐related AEs.^29^, ^30^, ^31^, ^32^, ^33^, ^34^, ^35^, ^36^ Teusink *et al*. reported no AEs (serious or otherwise) in 16 children with neuroblastoma, brain tumor, or relapsed malignancies aged 8 months to 15 years (median 6 years) who were receiving plerixafor,^28^ whereas Maschan *et al*. reported grade 1 or 2 AEs in eight of 33 (24.2%) children aged 1 to 18 years (median 9 years),^26^ and Sevilla *et al*. reported mild AEs in two of eight (25.0%) children aged 6–18 years (median 12.5 years).^27^ Although the latter two studies did not describe the age or underlying malignancies of the children with AEs,^26^, ^27^ the median age of the patients was older than that reported in the study by Teusink *et al*.,^28^ which may have contributed to the difference in AE rates between the studies. AEs reported in these studies included diarrhea, nausea, bone pain (possibly related to disease progression), pyrexia, and urticaria.^26^, ^27^ Similarly, some of the most common ADRs in our study were gastrointestinal events (vomiting, nausea, abdominal pain, and diarrhea).

Consistent with the findings of studies in adult Japanese patients with MM or NHL,^20^, ^21^ our study demonstrated that plerixafor is effective in achieving target CD34+ cell levels in Japanese children with solid tumors. Within 1 to 2 days of apheresis, almost 70% of our cohort of 18 pediatric patients achieved 2 × 10^6^ CD34+ cells/kg, which is the minimum recommended CD34+ cell level required for successful engraftment.^1^, ^2^ Other studies in pediatric patients who had failed previous mobilization with G‐CSF ± chemotherapy, or who had inadequate circulating CD34+ cell numbers to initiate apheresis, have demonstrated successful mobilization with plerixafor in ≥75% of patients.^25^, ^26^, ^27^, ^28^ In the MOZAIC study, significantly more children treated with plerixafor met the primary endpoint of doubling the CD34+ cell count in the 24 h before apheresis than those treated with standard mobilization alone, but high proportions of patients in both treatment arms reached ≥2 × 10^6^ CD34+ cells/kg within 1 to 2 days of apheresis (27/30 [94.4%] patients in the plerixafor arm and 15 of 15 [100.0%] patients in the standard treatment arm).^22^

Nakamura *et al*. have described their experience with pre‐apheresis CD34+ cell count as a predictor of adequate G‐CSF‐induced mobilization for A‐PBSCT in 42 Japanese children (≤17 years) with solid tumors.^37^ All the children with a peripheral blood CD34+ count of ≥10 cells/µL on the day before PBSC harvesting achieved the 2 × 10^6^ CD34+ cells/kg target on the day of harvesting, whereas 50% of patients with a CD34+ pre‐count of <10 cells/µL met this target.^37^ In the study of 33 pediatric patients who failed to achieve successful pre‐apheresis mobilization (defined as a CD34+ count of ≥20 cells/µL) after 4 days of G‐CSF administration, 93.9% of patients successfully mobilized CD34+ cells after treatment with plerixafor, with 81.8% of patients meeting the CD34+ cell target of 2 × 10^6^ cells/kg after one apheresis procedure.^26^ These findings suggest that a peripheral blood CD34+ cell count threshold of <10–20 cells/µL could be used to identify poor pediatric mobilizers who would benefit from the addition of plerixafor to standard mobilization. This should be studied in larger groups of patients.

Overall, our findings are limited by the uncontrolled, open label, PMS nature of the study. The small number of pediatric patients included in the study further limits the value of the current analysis. Also, no data were collected on the patients' history of previous A‐PBSCT or the rate of successful A‐PBSCT subsequent to plerixafor therapy.

### Conclusions

In summary, this study in 18 Japanese children with solid tumors who received plerixafor for HSC mobilization during routine clinical practice revealed no new safety concerns. ADRs, which occurred in seven patients (38.9%), were limited to the older patient subgroup (6 to <15 years), which may reflect different types of malignancies in older +versus younger children. Adequate CD34+ cell levels for harvesting were achieved within 1 to 2 days of apheresis in 12 of the 18 patients (66.7%). These data provide encouraging preliminary support for the effectiveness, safety, and tolerability of plerixafor at the internationally accepted dose of 0.24 mg/kg in Japanese children. The data also suggest that plerixafor may facilitate successful A‐PBSCT in Japanese children who are expected to be poor mobilizers. Further research in a larger cohort of patients with a wider range of malignancies, including lymphoma, is needed to verify this and to clarify the optimal timing of PBSC harvest and of CD34+ cell monitoring in peripheral blood.

## Disclosure

Hiroaki Goto has received fees for participation in review activities such as data monitoring boards from Sanofi K.K., Amgen K.K., Novartis Pharma K.K., Nippon Shinyaku Co. Ltd., Bayer Yakuhin Ltd., and Eli Lilly and Company; has received payment for writing or reviewing manuscripts from Nippon Shinyaku Co. Ltd.; has received payment for writing or reviewing manuscripts and lectures including service on speaker’s bureaus from Amgen K.K. Rie Kanamori and Takashi Seto are employees of Sanofi K.K. Satoshi Nishina is an employee of Sanofi K.K. and holds stocks in Sanofi. This study was funded by Sanofi.

## Author contributions

H.G. served as scientific advisor; S.N. contributed to study conception and design; T.S. contributed to enrollment of patients and operations; R.K. planned the pediatric subgroup analysis; all authors interpreted the results. All authors read and approved the final manuscript.
